# The complete mitochondrial genome sequence and phylogenetic analysis of yellow weasel (*Mustela sibirica*)

**DOI:** 10.1080/23802359.2019.1678432

**Published:** 2019-10-21

**Authors:** Mengshi Yu, Huailiang Xu, Diyan Li, Jiayun Wu, Anxiang Wen, Meng Xie, Qin Wang, Guangxiang Zhu, Qingyong Ni, Mingwang Zhang, Yongfang Yao

**Affiliations:** a College of Life Science, Sichuan Agricultural University, Ya’an, PR China;; b College of Animal Science and Technology, Sichuan Agricultural University, Chengdu, PR China

**Keywords:** *Mustela sibirica*, mitochondrial genome, phylogenetic analysis

## Abstract

In this study, we determined the complete mitochondrial genome sequence of the *Mustela sibirica.* The complete mitogenome of *M. sibirica* is 16,529 bp in length and consist of 13 protein-coding genes (PCGs), 2 rRNA genes, 22 tRNA genes, and a D-loop region. The overall base composition of the mitochondrial DNA is 32.88%A, 13.84%G, 27.32%T, and 25.96%C. The phylogenetic tree of the family *Mustelidae* constructed by using mitogenome sequences from 10 mustelid species of the family *Mustelidae*. These results provide necessary information for molecular phylogeny and evolutionary analysis of the *M. sibirica*.

Yellow weasel (*Mustela sibirica*) belongs to Carnivora, mustelidae. It is widely distributed in Siberia of Russia and China (IUCN 2016; Min 2018). Some weasels (*Neovison vison*, *Martes zibellina*, *M. sibirica*, etc.), are economic animal due to the value of the fur (Xu et al. 2010). In recent years, the aquaculture of the weasel is rising with the development of fur economic in China (Jian et al. 2012). In this study, we determined and analysed the complete mitochondrial genome of *M. sibirica*. This information will contribute to future phylogenetic studies of this species.

The specimen was collected from Ya’an (120°59′E, 29°58′N) and stored in Zoology laboratory of Sichuan Agricultural University, NO. 000756. The genomic DNA of *M. sibirica* was isolated from muscle tissue by using the TIANamp Genomic DNA extraction Kit. A previously published mitogenome of *M. sibirica* (HM106317) which downloaded from the National Centre for Biotechnology Information (NCBI) was used as a reference for gene annotation and primer design. The complete mitogenome sequence of *M. sibirica* which we determined was deposited in GenBank (No. MN264435).

The complete mitogenome of *M. sibirica* contains total 16,529 bp long, which consists of 13 protein-coding genes (PCGs), two rRNA genes (12S rRNA and 16S rRNA), 22 tRNA genes, and one D-loop region the same order as seen for other mammalian genes (Chun et al. 2010; Sang et al. 2017). Most PCGs are distributed on the H-strand, except for ND6 and eight tRNA genes which are encoded on the L-strand. The sequence is apparently AT biased (AT = 61.87%) with an overall base composition was 33.37% A, 28.50% T, 24.68% C, and 13.45% G. The longest PCG was the ND5 (1830 bp), whereas the shortest PCG was the ATP8 (204 bp). Ten PCGs use ATG as start codon, except for ND3 and ND5 initiate with ATA and ND2 begin with ATT. Of the 13 PCGs, the incomplete stop codons (T— or TA—) are used for termination of ND2, COX3, and ND4 (T—), ND1 and ND3 (TA–), respectively. AGA are used as stop codons in Cytb, and the other seven genes end with TAA. 12S rRNA and 16S rRNA are 959 and 1569 bp, respectively. These are located between tRNA^Phe^ and tRNA^Leu^ genes and are separated by the tRNA^Val^ gene. The 22 tRNA genes range in length from 62 (tRNA^Ser^) to 75 bp (tRNA^Leu^), tRNA^Ser^ has lost the stem of dihydrouridine (DHU) arm. The mitochondrial control region is 1121 bp long and is laid between the tRNA^Pro^ and tRNA^Phe^ genes.

The phylogenetic tree of the family *Mustelidae* constructed using the maximum-likelihood (ML) procedures implemented in MEGA version X (Kumar et al. 2018). To further explore, the taxonomic status of the *M. sibirica* and its phylogenetic relationship within the family *Mustelidae*, a phylogenetic tree constructed using mitogenome sequences from 10 mustelid species of 3 genera (Figure 1). According to the phylogenetic tree, *M. sibirica* clustered into a clade with *M. nigripes* and *M. putorius* and all the members of the genus *Mustela* form a monophyletic group. In all, this genome will provide a molecular basis for the conservation and research of *M. sibirica*.

**Figure 1.**
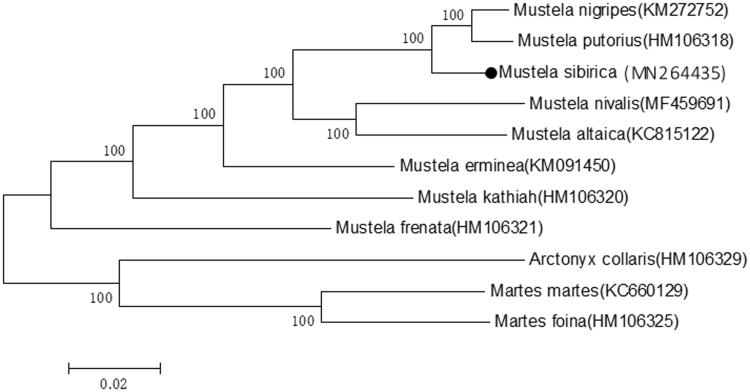
Neighbour-Joining (NJ) phylogenetic tree constructed based on mitogenome sequences from 11 mustelid species of 3 genera. Numbers at the branches indicated the bootstrapping values with 1000 replications. GenBank accession numbers are given in the parentheses. Filled circle represented a sequence from this study.
